# Renal epithelial and stromal tumor with a multiple cystic lesion localized in the upper portion of the right kidney

**DOI:** 10.1007/s13730-020-00548-9

**Published:** 2020-11-03

**Authors:** Masato Sawamura, Naoki Sawa, Masayuki Yamanouchi, Daisuke Ikuma, Akinari Sekine, Hiroki Mizuno, Tatsuya Suwabe, Junichi Hoshino, Kei Kono, Keiichi Kinowaki, Kenichi Ohashi, Yoji Nagashima, Yoshifumi Ubara

**Affiliations:** 1 Nephrology Center and Department of Rheumatology, Toranomon Hospital Kajigaya, 1C-1, Takatsu, Kawasaki, Kanagawa 212-0015 Japan; 2 Department of Pathology, Toranomon Hospital, Tokyo, Japan; 3 Okinaka Memorial Institute for Medical Research, Toranomon Hospital, Tokyo, Japan; 4 Department of Surgical Pathology, Tokyo Women’s Medical University, Tokyo, Japan

**Keywords:** Unilateral renal cystic disease, Multilocular cystic nephroma, Renal epithelial and stromal tumor, Autosomal dominant polycystic kidney disease

## Abstract

A 60-year-old Japanese woman was admitted because of the polycystic mass with right flank pain localized in the upper portion of the right kidney. Right nephrectomy was performed because the mass lesion had continuously increased in size over the past 10 years. A surgical specimen showed histology consistent with a mixed epithelial and stromal tumor, which is closely related to multilocular cystic nephroma, and was diagnosed by a defined capsule between the cystic mass lesion and normal renal tissue by CT and MRI, and histology. Localized renal cystic disease that does not have a capsule was excluded from differential diagnosis.

## Introduction

Localized renal cystic disease (LRCD) has been reported; cysts form in only one kidney and not in other organs, and patients do not have a family history of cystic disease, unlike autosomal dominant polycystic kidney disease (ADPKD). Two types of LRCD have been reported: segmental renal cystic disease, which involves just one part of a kidney, and unilateral renal cystic disease (URCD), which involves a whole kidney [1, 2]. Here, we encountered a polycystic mass only in the upper portion of the right kidney in a 60-year-old female patient, and URCD/LRCD has been considered first diagnosis, but finally localized multilocular cystic nephroma (MCN) became definitive diagnosis. Differential diagnosis of two type of polycystic diseases including URCD/LRCD and MCN will be discussed clinically, pathologically and by diagnostic image on this issue.

## Case report

A 60-year-old Japanese woman was admitted to our hospital for evaluation of a polycystic mass in the upper portion of the right kidney and right flank pain. The mass lesion was first identified when the patient was aged 50 years, and regular computed tomography (CT) scans had shown that the lesion had gradually enlarged over time. The patient had no cysts in the liver, left kidney, or the remaining area of the right kidney. This patient does not have a history of long-term estrogen replacement.

On admission, the patient was 154 cm tall and weighed 70 kg. Her blood pressure of 150/82 mmHg; and her temperature, 36.6 °C. She had a history of hypertension, dyslipidemia, hyperuricemia, primary hyperparathyroidism, and Graves’ disease, but she had no family history of cystic disease. Laboratory findings were as follows: white blood cell count, 7000 /μL; hemoglobin, 14.0 g/dL; platelet count, 30.2 × 10^3^/μL; total protein, 7.2 g/dL; albumin, 4.4 g/dL; serum urea nitrogen, 27 mg/dL; serum creatinine, 1.3 mg/dL; and estimated glomerular filtration rate(eGFR), 35.0 ml/min/1.73 m^3^. Urinary protein excretion was 0.1 g/day and the urinary sediment contained no erythrocytes or casts.

## Radiological diagnosis

**Fig. 1:**
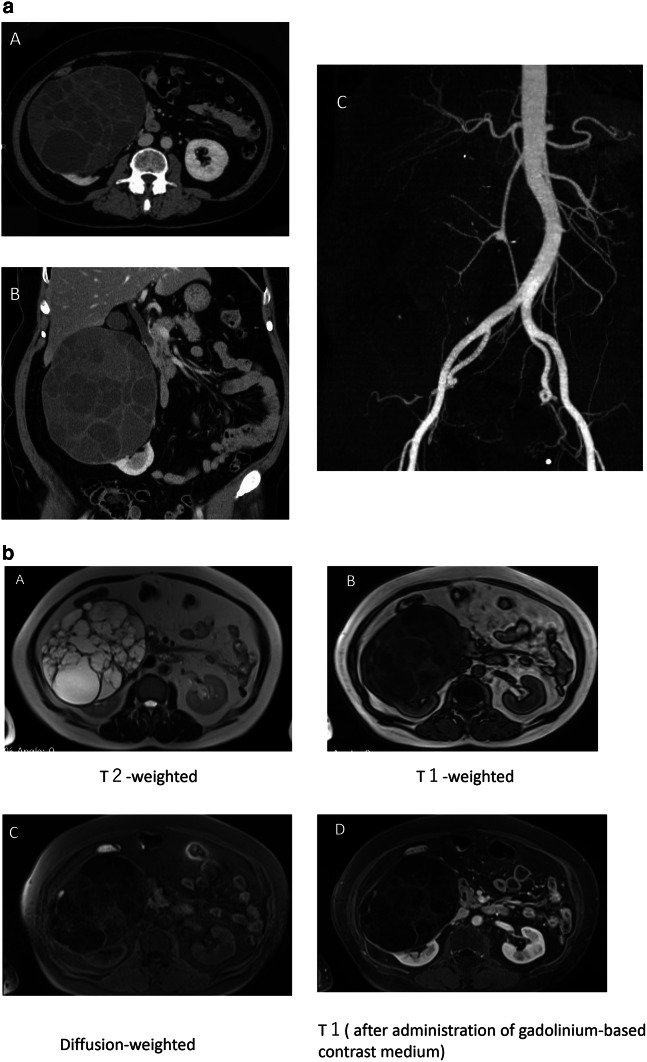
**a** Computed tomography (CT) showed a polycystic mass in the upper portion of the right kidney measuring 17 × 15 × 12 cm (A: axial section, B: coronary section). CT angiography (C) revealed large, elongated, well-developed renal arteries surrounding the large mass lesion but did not detect any hypervascular stain, i.e. there was no indication of a malignant tumor. The abdominal aorta was shifted and elongated towards the left as a result of the polycystic mass. **b** Magnetic resonance image (MRI) showed hyperintensity on the T2-weighted images (A) and normality or hypointensity on the T1-weighted (B) and diffusion images (C). T1 (after administration of gadolinium-based contrast medium (D)

CT showed a polycystic mass in the upper portion of the right kidney that measured 17 × 15 × 12 cm and had well-defined margins. CT angiography revealed large, elongated, well-developed renal arteries around a large mass lesion but did not detect any hypervascular stain, i.e. it did not indicate the presence of a malignant tumor. The abdominal aorta was shifted and elongated towards the left as a result of the polycystic mass (Fig. 1a).

The mass lesion was hyperintense on the T2-weighted MRI images and normal or hypointense on the T1-weighted and diffusion images (Fig. 1b; this finding was inconsistent with ADPKD, which usually consists of mixed cysts that are hyperintense and hypointense on MRI. MRI confirmed that the lesion had well-defined margins. In a 99mTc-diethylenetriaminepentacetate (DTPA) scan, the glomerular filtration rate of the right kidney was 16.9 mL/min; and of the left kidney, 39.3 mL/min.

URCD/LRCD, localized multilocular cystic nephroma (MCN), multicystic dysplastic kidney, and low-grade cystic renal cell carcinoma were proposed as possible differential diagnoses.

## Renal histology

Our nephrology team decided to remove the right kidney because the mass lesion had continuously increased in size over the past 10 years and the patient was experiencing pain.

**Fig. 2:**
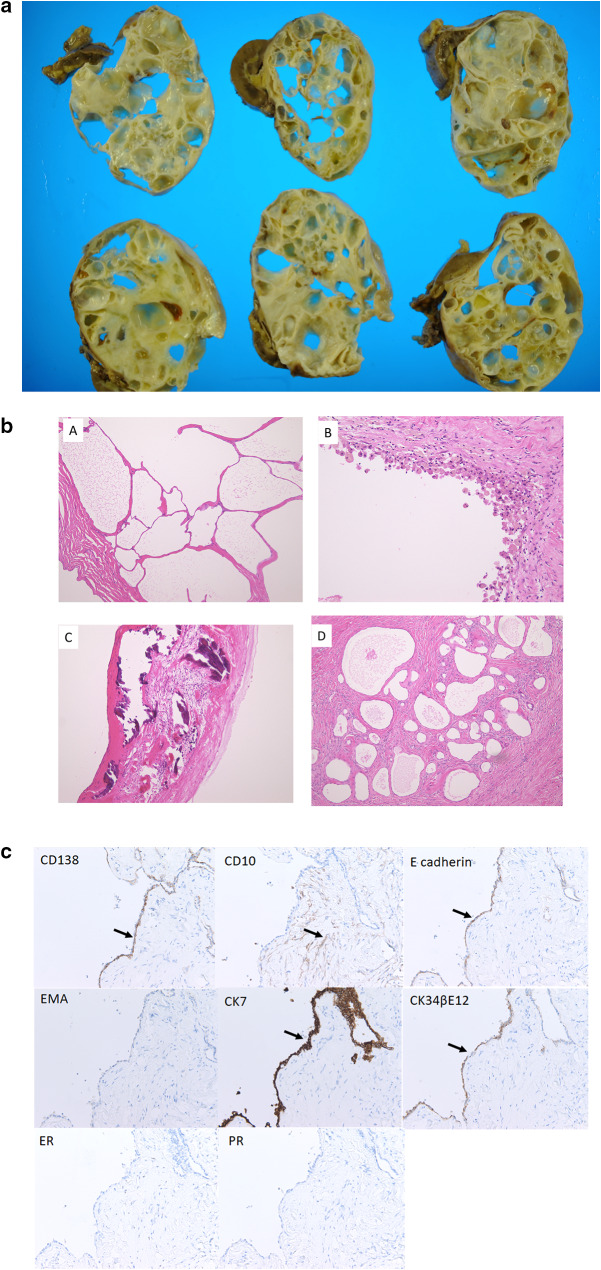
**a** Macroscopic examination of the mass lesion (size: 17 × 15 × 12 cm). **b** Microscopic examination of the mass lesion showed a cystic lesion with a thickened cystic wall and mostly flat or cuboidal single-layer epithelium (A); hobnail epithelium (B) and calcification (C) were also noted. Stroma consisting of collagen fiber-rich lesion with spindle cells similar to ovarian interstitial tissue was noted between the cysts (D). **c** The epithelial component was positive for CD138, E-cadherin, CK7, and CK34βE12 (brown coloring, arrow) but negative for CD10, epithelial membrane antigen (EMA), estrogen receptors (ER), and progesterone receptors (PR). The stromal element was positive for CD10 (arrow) but negative for CK7, ER, and PR

Macroscopic examination of the resected kidney showed a polycystic mass measuring 17 × 15 × 12 cm (Fig. 2a), and microscopic examination revealed a fibrous capsule forming a border between the lesion and the normal renal tissue (Fig. 2a). The lesion consisted of the cystic mass with a thickened cystic wall and mostly flat or cuboidal single layer epithelium (Fig. 2b-A); hobnail epithelium (Fig. 2b-B) and calcification (Fig. 2b-C) were also noted. Stroma consisting of a collagen fiber-rich lesion with spindle cells similar to ovarian interstitial tissue was present between the cysts (Fig. 2b-D). We did not find glomeruli, renal tubules, or hemorrhage in the septa between the cysts and did not detect malignant cells. The pathological findings were compatible with a diagnosis of mixed epithelial and stromal tumor (MEST) [3–7].

## Immunohistochemical analysis

We performed immunohistochemical analysis to investigate whether the cystic epithelial and stromal component had the characteristics of each of the three parts of normal renal tubules. We chose CD138 and CD10 as markers of the proximal renal tubule; E-cadherin, epithelial membrane antigen (EMA), and CK7 as markers of the loop of Henle and the distal convoluted tubule; and CK34βE12 as a marker of the collecting duct [3]. Estrogen receptors (ER) and progesterone receptors (PR) were stained according to previous reports of MEST [4–7].

The epithelial component was positive for CD138, E-cadherin, CK7, and CK34βE12 but negative for CD10, EMA, ER, and PR. The stromal element was positive for CD10 but negative for CK7, ER, and PR (Fig. 2c).

We decided that the most reliable diagnosis in our patient was MCN/MEST/ renal epithelial and stromal tumor (REST) but not LRCD.

## Further clinical course

Nine years after nephrectomy, the patient still has good renal function, with a creatinine level of 1.53 mg/dL and eGFR of 27.8 ml/min/1.73 m^3^.

## Discussion

We encountered a case of MCN/MEST/REST but not LRCD. Information necessary for differential diagnosis of these disease was examined. Slywotzky et al. summarized 18 reported cases of URCD (15 men, three women) [1]. The patients’ median age at diagnosis was 50 years (range 24–83 years). Five patients presented with hematuria, four with flank pain, and one with a palpable abdominal mass; the disease was an incidental finding in eight patients. The researchers described URCD as a non-familial, non-progressive disease that is not associated with kidney failure and does not present with cysts in other organs [1]. They wrote that URCD may be confused with ADPKD because the gross and histological findings may be similar, but none of the 18 patients had a family history of ADPKD [1]. Dowden et al. presented a case of LRCD and described the specific characteristics in MRI and surgical histology [2]: MRI showed that the cystic mass lesion did not have any capsule at its border with normal renal tissue, and histological findings showed normal kidney parenchyma, including glomeruli and tubules between the cysts [2].

In the differential diagnosis of URCD, physicians also need to consider multilocular cystic nephroma (MCN). MCN is a rare, benign cystic lesion of the kidney with a bimodal age distribution, i.e. it occurs in both infants and adults. Patients usually present with nonspecific symptoms, but abdominal pain, hematuria, and urinary tract infection are common in adults. CT shows a multicystic architecture with well-defined margins. MRI usually shows a hypointense signal on T1-weighted sequences and a hyperintense signal on T2-weighted sequences [8].

According to the World Health Organization classification of renal neoplasms, adult-type MCN is grouped histologically with the mixed epithelial and stromal tumors (MEST) [4]. The term renal epithelial and stromal tumor (REST) can be used to encompass both MCN and MEST [5]. MEST, a complex solid and cystic renal tumor with stromal and epithelial elements, occurs almost exclusively in perimenopausal women [5–7]. The stromal component consists of spindle cells that mimic ovarian stroma and express ER and PR, as well as CD10. The epithelial component contains epithelium-lined cysts or microcysts and may be positive for CK 7 [6, 7]. In our patient, the stromal component of the lesion was not positive for ER and PR specific to MEST, but the epithelial component was positive for CD138, E-cadherin, CK7, and CK34βE12. These findings indicate that the polycystic mass in our patient may have been closely related to any substrate common to all segments of the renal tubules [3]. Caliò et al. evaluated MEST of 53 Cases, and found that stains for PR and ER showed positivity in the stromal component in 85 and 73% of stromal component [9]. Since patients with MEST of the kidney was predominant in females with a history of long-term estrogen replacement, ER and PR staining was applied to immunohistochemical staining of patients with MEST [10]. However, this patient did not have the history of long-term estrogen replacement. This may indicate the reason why the component was negative for ER and PR. In addition, the positivity of ER and PR staining test has been considered an indicator of the growth of several cancers including breast cancer and the response to the endocrine therapy. Our case of MEST may show the neoplastic lesion which has low grade [11].

In conclusion, the surgical specimen of this patient’s polycystic mass localized in the upper portion of the right kidney diagnosed MEST histologically. MEST is close related to MCN, which has been confirmed to have a defined capsule between the cystic mass lesion and normal renal tissue by CT and MRI, and by renal histology.

## Abbreviations

ADPKD: Autosomal dominant polycystic kidney disease
CT: Computed tomography
DTPA scan: Diethylenetriaminepentacetate scan, LRCD, localized renal cystic disease
MCN: Multilocular cystic nephroma
MEST: Mixed epithelial and stromal tumor
MRI: Magnetic resonance imaging
REST: Renal epithelial and stromal tumor
URCD: Unilateral renal cystic disease
EMA: Epithelial membrane antigen
ER: Estrogen receptors
PR: Progesterone receptors
VATS: Video-assisted thoracoscopic surgery

## References

[1] Slywotzky CM, Bosniak MA (2001). Localized cystic disease of the kidney. Am J Roentgenol.

[2] Dowden EE, Osunkoya AO, Baumgarten DA (2010). Localized cystic disease of the kidney: an unusual entity that can mimic a cystic neoplasm. Am J Kidney Dis.

[3] Bonsib SM, Jennette JC, Olson JL, Silva FG (2015). Renal anatomy and histology. D9Agati VD Heptinstall’s pathology of the kidney.

[4] Humphrey PA, Moch H, Cubilla AL, Ulbright TM, Reuter VE (2016). The 2016 WHO Classification of tumours of the urinary system and male genital organs-part b: prostate and bladder tumours. Eur Urol.

[5] Turbiner J, Amin MB, Humphrey PA, Srigley JR, De Leval L, Radhakrishnan A, Oliva E (2007). Cystic nephroma and mixed epithelial and stromal tumor of kidney: a detailed clinicopathologic analysis of 34 cases and proposal for renal epithelial and stromal tumor (REST) as a unifying term. Am J Surg Pathol.

[6] Chu LC, Hruban RH, Horton KM, Fishman EK (2010). Mixed epithelial and stromal tumor of the kidney: radiologic-pathologic correlation. Radiographics.

[7] Zheng S, Yuan HC, Liu LR, Wei Q, Han P (2013). Mixed epithelial and stromal tumor of the kidney. Kaohsiung J Med Sci.

[8] Wilkinson C, Palit V, Bardapure M, Thomas J, Browning AJ, Gill K, Biyani CS (2013). Adult multilocular cystic nephroma: report of six cases with clinical, radio-pathologic correlation and review of literature. Urol Ann.

[9] Caliò A, Eble JN, Grignon DJ, Delahunt B (2016). Mixed epithelial and stromal tumor of the kidney: a clinicopathologic study of 53 cases. Am J Surg Pathol.

[10] Adsay NV, Eble JN, Srigley JR, Jones EC, Grignon DJ (2000). Mixed epithelial and stromal tumor of the kidney. Am J Surg Pathol.

[11] Elledge RM, Green S, Pugh R, Allred DC, Clark GM, Hill J, Ravdin P, Martino S, Osborne CK (2000). Estrogen receptor (ER) and progesterone receptor (PgR), by ligand-binding assay compared with ER, PgR and pS2, by immuno-histochemistry in predicting response to tamoxifen in metastatic breast cancer: a Southwest Oncology Group Study. Int J Cancer.

